# Moral injury and burnout among saudi educators under vision 2030: An interpretative phenomenological study of systemic ethical tensions

**DOI:** 10.1371/journal.pone.0356254

**Published:** 2026-08-14

**Authors:** Mazin Mansory

**Affiliations:** English Language Institute, King Abdulaziz University, Jeddah, Saudi Arabia; UTM Skudai: Universiti Teknologi Malaysia, MALAYSIA

## Abstract

Moral injury is a term that refers to the psychological and emotional impact of an individual’s actions, or inactions, which violate their ethical standards and beliefs. This study examined how educators working at educational institutions in Saudi Arabia are experiencing both moral injury and burnout due to the increased reliance on standardized performance assessment and increased bureaucracy associated with the implementation of Vision 2030. In-depth semi-structured interviews were conducted with 25 educators representing a range of curriculum areas and locations throughout Saudi Arabia. The interview data were analysed using Interpretative Phenomenological Analysis (IPA). Six major themes emerged: systemic pressures, curriculum rigidity, reduced relational care, value dissonance, burnout, and lack of institutional empathy. Interviewees frequently expressed feelings of ethical betrayal and spiritual pain that occurred when they had to comply with standardized mandates that contradicted the Islamic pedagogical values of care, justice, and moral guardianship. Furthermore, there were generational differences noted: new teachers reported acute identity insecurity, while experienced teachers appeared to compartmentalize their teaching activities and develop what is referred to as “dual teaching”. These findings suggest how moral injury theory may be extended to non-Western and religiously based contexts and illustrate the importance of implementing reform in a way that is relationally sensitive to the ethical commitments and cultural frameworks of educators.

## Introduction

As part of its 2030 Vision, Saudi Arabia is trying to improve education by using the latest technology, performing better in the world, and providing excellent educational services to citizens [1]. As part of this initiative, teachers are considered the key drivers of change because they are responsible for implementing technology into education, following a standardised way of teaching, and using an outcome-based approach in the classroom [2–4]. Throughout the world, across numerous nations, many of the same strategies for coping with teacher burnout have resulted in increased workloads for teachers, surveillance and auditing cultures within schools, and increased levels of job-related stress or teacher burnout [5]. Scholars recently have identified a new type of trauma that they believe should be included in consideration for educators: moral injury [6,7]. The term ‘moral injury’ has been defined as an injury to one’s spiritual and psychological wellbeing as a result of performing an action contrary to one’s deeply held moral beliefs, or when a person discovers that they believe their institutional or professional framework has betrayed their professional values [8]. The use of moral injury in education occurs when educators experience guilt over participating in an action that has a negative effect on a student or students, compromises their pedagogical integrity or violates their religion [9]. This is an important distinction, as while burnout is a reflection of emotional depletion, moral injury represents a breakdown in ethical identity and purpose as an educator.

In order to firmly establish the study’s content in clearly defined constructs, three conceptually similar but analytically differentiated constructs have been identified for this project. Moral injury refers to the mental and spiritual damage done when forced into action that runs contrary to one’s ingrained beliefs or when an individual feels that their institution has violated the ethics that they were taught to trust. This includes an impact caused by a transgressive act (either one that was committed, witnessed, or an act an individual did not have enough strength to thwart), the sense of a violation regarding one’s religious/moral code, and the potential effects on the individual’s moral identity, such as resulting feelings of shame, guilt, betrayal, and fragmentation of one’s identity [10,11]. According to Maslach and Jackson [12], burnout is a syndrome that consists of three components that represent the chronic depletion of resources: emotional exhaustion, depersonalization (detachment and disregard), and reduced sense of personal accomplishment. There is also a chronic occupational demand associated with each of these burnout dimensions, which does not require a precipitating moral infraction. Teacher demoralisation phenomena is when teachers become less committed to their profession over time due to being prevented from doing the type of work they believe is morally right by the system that governs their work [13]. Teacher demoralisation occurs specifically when conditions within a school or organisation make it impossible for teachers to do good work. Teacher demoralisation may occur along with other constructs, such as moral injury (which occurs in very specific situations) or burnout (which happens when a teacher runs out of resources), but these constructs are different in nature. In this study, moral injury was treated as the main construct for analysis, while also considering how it interacts with burnout and demoralisation, as outlined in the stories that each participant has shared.

The distinction between burnout and moral injury is significant in Saudi Arabia because education is viewed as more than just a way to impart knowledge and develop skills; it is also viewed as a moral vocation founded on Islamic principles of responsibility, justice, and caring for others [14]. The Vision 2030 plan incorporates several neoliberal governance theories, such as Key Performance Indicators (KPIs), pacing mandates, and a culture of inspection, which tends to promote compliance over contextual responsiveness [15]. When these governance mechanisms hinder teachers from acting according to their religious and ethical beliefs, they may experience professional distress due not only to stress but also to their sense of moral transgression and spiritual conflict. Although global interest in the well-being of teachers’ lives under accountability systems has increased significantly, limited research has been conducted to understand how non-Western, religiously based systems of education experience the ethical consequences of reform. The majority of research conducted to date in Saudi Arabia has framed the development of teachers in terms of the skills they need to develop and prepare for the use of technology or for implementing the policies being implemented at the time. Researchers have focused very little attention on how teachers engage in moral reasoning, how reform influences their sense of identity, and how their own spiritual lives are disrupted through their profession [16]. Therefore, there is little understanding of how systematic reform affects teachers’ ethical conflicts in the classroom or how those conflicts develop over time.

The research gap addressed by this study is therefore threefold and can be stated precisely. First, there are no published Interpretative Phenomenological Analysis studies examining moral injury among teachers in Islamic educational systems, leaving a methodological absence at the intersection of idiographic qualitative inquiry and religiously grounded professional ethics. Second, existing ecological accounts of teacher wellbeing have not been empirically developed for Gulf-state reform contexts, where the interaction of rapid centralized reform with established religious-pedagogical traditions is structurally distinct from the Anglophone settings that dominate the literature. Third, religious ethics as a mediating variable in moral injury scholarship remains systematically under-theorized, despite its centrality to teachers’ professional identity in much of the world. This study addresses all three dimensions of this gap by combining IPA with an ecologically grounded analysis of moral injury in three purposively selected Saudi regions.

To fill this gap, this research explored how teachers in Saudi Arabia experience both moral injury and burnout through the ecology of Vision 2030 reforms. By using Bronfenbrenner’s ecological systems model of development to study the interaction of macro-level policy with institutional frameworks and individual teachers’ experiences, this research provided an understanding of how these reforms affect teachers’ ethical identities and well-being. By doing so, this research contributed to developing a culturally based ecological model of moral injury in education, as well as extending the existing literature beyond mostly Western and secular contexts.

### Rationale

The primary reason for undertaking this study is because there is a paucity of research studies into moral injury in education. Although the volume of research being conducted into moral distress (teachers) and teacher demoralization is increasing, it is still mostly being carried out in western secular policy environments where ethical harm is conceptually understood through either psychological or organizational lenses [17]. There is little understanding of how moral injury occurs in educational environments where ethical responsibility is both a professional responsibility, as well as a cultural expectation, spiritual obligation and expectation. The Saudi educational system represents an increasingly theoretically important example of how teachers view transgression as both a professional violation and a rupturing of the spirit [18]. The rapid reforms of the Saudi educational system under Vision 2030 has thrown these newly transformed educational systems which have been “remade” by centrally mandated accountability systems, pacing systems, and performance – based evaluation of teacher performance into sharp relief [19]. However, this regime of governance of the very background forms of organization and responsibility that the above listed resemble so much the roll out of education reforms under neoliberalism internationally, the social conditions of the workplace of the teacher are utterly different because teaching as understood in traditional ways (*Muraqib*) as a form of moral stewardship and public (community) obligation.

The intersection of those two realities can be an experience of complicity in the demands of reform rather than technical readjustment and can be either an ethical betrayal or ethical harm, allowing moral injury to be a culturally mediated experience leading to systemic contradiction rather than the vulnerability of individual teacher. Recent studies conducted on the national level suggest that many teachers in Saudi Arabia are becoming burnt out, primarily in the areas of the demands for integrating inclusive education and digitalization of curriculum. But teachers’ experiences cannot be accounted for in isolation by burnout experiences. Many teachers describe their experience as a phenomenon of a disruption of their sense of identity, spiritual conflict and ethical betrayal. Therefore, our aim is to take beyond the current research that is limited to a deficit-based model of how the wellbeing of teachers and explore the ethics-structural dimensions of teacher distress in the context of educational reforms can be restored. This qualitative study used interpretative phenomenological analysis (IPA) to allow for an examination of teachers’ sense-making, reflection and internalization of ethical dilemmas in their lives. To gain insights on how the policies enacting educational reform are interpreted and acted upon in local communities, data was collected from a national sample of Saudi educators recruited across multiple regions of the Kingdom, with three primary anchor sites in Riyadh, Jeddah and Abha, to obtain a holistic view on the dynamics of how the educational reforms are taking place at different ecological levels of implementation in terms of central authority and community embeddedness. This multi-site analysis provides an ecological framework for this research in terms of the varying dynamics of how teachers, school, and community mediate the effects of an educational reform, set within the local sociocultural or environmental context. By situating the issue of moral injury within the interplay of policy, institution and classroom environment, this research contributed to an ecological understanding of ethical harm within education and expanded literature related to moral injury to non-Western based education reform systems.

### Theoretical framework

The approach of this article combines moral injury theory and Bronfenbrenner’s ecological systems framework to view and understand teachers’ ethical distress as a process occurring within a system and through development rather than as an individual psychological shortcoming. Moral injury refers to psychological and spiritual harm that occurs when individuals are compelled to act against deeply held moral commitments or experience institutional betrayal of professional values [10,11].

In educational settings, this harm often arises when bureaucratic requirements constrain ethical pedagogy and relational responsibility [9,13,20]. While theoretically useful in addressing the internal experience of ethical transgression, the theory is silent about how structures create this distress; Bronfenbrenner’s ecological systems theory fills this in by modelling how macrolevel policies are operationalized through layers of institutional structure to experience as classroom conditions.

By integrating these two theories, this study develops an interpretive ecological framing of moral injury where ethical harm emerges from a three-stage process. First is translation, a process where national reform agendas become converted into the institutions’ expectations, performance indicators, and compliance routines (macrosystem to exosystem). Second is a collision where these demands enter the classroom microsystem, conflicting with educators’ moral, values-based pedagogical and relational commitments, including Islamic precepts of care, justice, and moral stewardship that govern teacher-student relations. The third stage of internalization comes with repeated experiences of ethical compromise that become psychologically and spiritually internalized as moral injury (betrayal, fragmented self, loss of professional meaning). In this context, burnout is understood not as the etiology of moral injury but rather as a strain response to the moral-structural work of the reform system.

To clarify the status of this model: it is presented here as a theoretically informed framework that was refined iteratively against participants’ narratives rather than as a hypothesis tested against pre-specified measures. Bronfenbrenner’s ecological layers and Litz et al. [10] moral injury components functioned as sensitizing concepts during idiographic analysis; the three processual stages, Translation, Collision, and Internalization, emerged abductively as the most parsimonious account of how macrosystem reform demands become phenomenologically registered as moral injury in participants’ accounts. Translation denotes the institutional conversion of macrosystem policy (Vision 2030, global accountability ideology) into exosystemic demands on teachers, Ministry directives, inspection cycles, Key Performance Indicator frameworks, and pacing mandates. Collision denotes the encounter, at the microsystem of the classroom, between these translated demands and teachers’ culturally and religiously grounded pedagogical commitments (compassionate care: ‘*Ra’hmah*’; justice: ‘*Adl*’; moral stewardship: ‘*Muraqib’*). Internalisation denotes the individual teacher’s phenomenological registration of repeated collisions as moral injury, shame, guilt, ethical betrayal, and fragmentation of professional identity, moderated across the chronosystem by career stage.

The model also accounts for the variation across career stages (chronosystem). Early career teachers with less institutional power and professional capacitation may experience moral injury as deep, acute insecurity in their identity and role, whereas veteran teachers may cope in more adaptive but ethically costly ways, e.g., as compartmentalizer or two-faced. Effects of chronosystem thus moderate the experience and expression of moral injury. This integrated framework inputs into guiding research questions and analytic strategy and technique by tying the politics of reform systems into the lived ethical experience.

**Fig 1:**
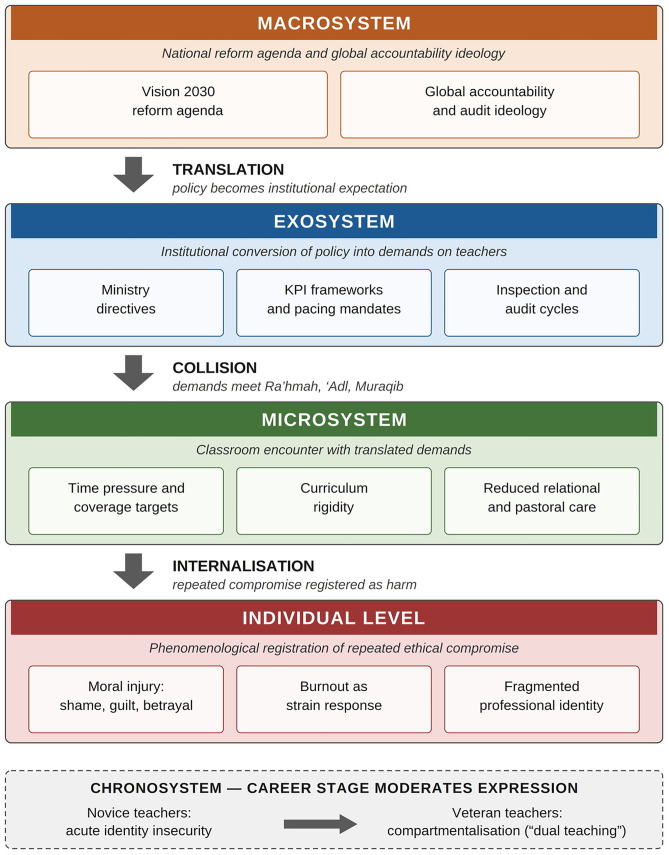
The interpretive ecological moral injury framework.

Fig 1 highlights a three-stage process where education reform agendas and accountability ideology (macrosystem) are operationalized through Ministry directives, inspection regimes, and performance indicators (exosystem), creating classroom conditions of time pressure and reduced relational care (microsystem), and ultimately internalised as moral injury at the individual level, with chronosystem accounting for age differences in experience and expression between novice and veteran teachers.

Through the evaluation of alternative theoretical lenses in relation to the contextualisation of the framework selection process, both organisational stress and job demands–resources [21] effectively explain resource depletion processes; however, they do not incorporate the ethical-identity rupture that participants described. Role conflict theory identifies structural incompatibility of roles but does not address the moral components of that incompatibility. Self-determination theory discusses loss of autonomy; however, it does not provide context for the sacred and/or religious basis for the pedagogical commitment in Islamic educational settings. Moral injury theory was retained as the primary lens as it alone highlights the transgressional nature of the ethical interaction and the spiritual aspects of the harm caused. The ecological framework was added to mitigate the lack of moral injury theory’s attention towards the structural causes of these types of interactions.

### Research questions

This research study addressed three main research questions:

1. How do Saudi educators experience and interpret moral injury within the context of Vision 2030 educational reforms?
2. How do institutional, cultural, and systemic conditions contribute to educators’ experiences of moral conflict and ethical dissonance?
3. How do teachers navigate, negotiate, or resist ethical tensions generated by reform-related accountability demands across different career stages?

### Literature review

1. Conceptual Foundations of Moral Injury in Education

Moral injury originated in military psychology and referred to psychological and spiritual distress resulting from actions, or inaction, that violate deeply held moral beliefs [10,11]. This construct has since been applied to health, social work, and educational settings, where professionals may suffer harm when institutional imperatives diverge from moral obligations to student-centred care [13,22]. In education, moral injury emerges from pressuring teachers into compliance with policies that violate pedagogy and child welfare, resulting in resentment and betrayal, accountability demands, and rupture of professional and social identity [7].

While burnout is essentially an expression of exhaustion and depersonalisation from exposure to excessive labour over time, moral injury is predicated upon violation and dissonance of value [23,24]. It is through this lens that educators were recruited in a study of distress under reform in Saudi Arabia, treating their suffering not as fatigue or overwork but as conflict over constrained moral agency.

2. Global Research on Teacher Moral Distress and Reform

Internationally, accountability driven reforms in systems also emphasising high stakes testing, performance monitoring, and datafication have been linked to moral distress amongst teachers [25]. Santoro [13] has coined the term teacher demoralization to make the case for educators leaving the profession not because of heavy workload per se but because they are prevented from doing the things they know are morally right. Empirical studies in Australia and the UK and France similarly identify institutional betrayal and ethical compromise as drivers of professional disillusionment [9,26,27]. However, generally the literature has its origins in secular western policy environments, so less attention has been paid to the implications of moral injury in educational systems in which teachers work within denser religious and community ethics, which creates a significant gap in moral injury scholarship.

3. Gaps in Non-Western Educational Contexts

Research on education in the Middle East has historically neglected to tease out the ethical and spiritual aspects of teaching as a practice under reform [28], opting instead to frame pedagogical practice predominantly in terms of structural conditions like salaries, facilities, and professional development. However, in many Arab contexts teaching is socially constructed simultaneously as a profession and as a moral vocation, drawing on religious ideas of stewardship (*Muraqib*) and the principle of communal responsibility. As a result, the omission of moral analysis from reform research leaves unexplained how teachers come to experience moral conflict within religious systems of knowledge. This gap is particularly salient for fast reforming societies like Saudi Arabia, where global accountability models are rapidly grafted onto traditions resting on bedrocks of values.

4. Vision 2030 and the Saudi Educational Reform Landscape

Vision 2030 reforms have encompassed curriculum modernization, digitization, standardization, assessment, English as the medium of instruction, closer university-business connections, and educational -labour market alignment [29,30], are inspired by neoliberal governance logics of efficiency, productivity, evaluation, and accountability in terms of results and outcomes. Teachers are to be governed through key performance indicators (KPIs) related to test results, attendance, and pacing in curriculum coverage [31]. The early research record suggests increasing professional anxiety associated with a growing inspection regime and anxieties around “failure” of compliance within imposed hierarchical structures [32]. Within this changing ethically fraught context of education it may be harder to exercise pedagogical discretion and offer loving relational care, creating ethical dilemmas for educators in the minutiae of everyday classroom life.

5. Theoretical Anchoring: Moral Injury Meets Ecological Systems

Moral Injury Theory explains how the individual sees themselves after inflicting ethical harm on others or potentially being harmed by others and provides the basic premise for understanding Ecological Systems Theory [33,34]. When used in concert, both frameworks provide clear insights regarding the macro-level reform agenda, and how that macro-level reform agenda is translated to teachers at the school-level through exosystemic institutions, such as the workplace, which creates the very system of ethical tension teachers experience every day [33,34].

This integrated perspective enables analysis of how systemic accountability reforms may erode teachers’ moral agency and professional identity over time, particularly within culturally and religiously grounded educational contexts. By situating moral injury within interacting ecological layers, the present study offers a structurally grounded explanation of ethical distress rather than attributing teacher suffering to individual resilience deficits.

To clarify the conceptual progression and thematic coverage of the literature review, Table 1 summarizes the major sections, focal constructs, and representative sources that informed the theoretical and empirical foundations of this study.

**Table 1:** Structure of the literature review.

Key References	Description	Section
Shay [11], Litz et al. [10], Santoro [13]	Definition of moral injury and moral injury as a research term, from military psychology to educational literature and practice. Establishes that moral injury refers to ethical and emotional damage caused by a disconnect between policy and practice.	2.1 Moral Injury in Education
[12], Taris et al. [35]	Summary of the global literature on teacher burnout as a social phenomenon, including symptoms, causes, consequences associated with teacher burnout (with a noted emphasis on emotional exhaustion), depersonalization and reduced personal accomplishment.	2.2 Teacher Burnout and Emotional Exhaustion
Elyas and Picard [36], Wiseman [37]	Overview of the literature describing educational policy reforms, neoliberal accountability constructs and standardization in the K-12 educational system in Saudi Arabia framed in the context of the Kingdom’s Vision 2030 reform agenda; including evidence of systemic tensions in education in the English language.	2.3 Educational Reform and Systemic Pressures in Saudi Arabia
Al-Sadan [38], Smith and Abouammoh [39]	Overview of Islamic pedagogy, cultural expectations and educational values based in religious beliefs shaping teachers’ professional identities.	2.4 Cultural and Religious Contexts of Teaching in the Kingdom
Santoro [13], Jennings and Greenberg [40]	Summary of some of the research connecting moral injury and burnout to show how dissonance contributed to increased emotional exhaustion and exhaustion of identity.	2.5 Intersection of Moral Injury and Burnout
Author’s own synthesis: multiple gaps identified	Briefly identifies some empirical and theoretical gaps in the current literature, specifically a lack of studies related to moral injury in a non-Western and specifically Islamic educational context.	2.6 Gaps in the Current Literature

## Methodology

### Research design and conceptual framework

This study employed an interpretative phenomenological analysis (IPA) design to examine how Saudi educators experience and interpret moral injury and burnout within the context of Vision 2030 reforms. IPA is grounded in Heideggerian phenomenology and is concerned with how individuals make sense of emotionally complex and ethically charged experiences [41,42]. Given that moral injury involves violations of deeply held moral commitments rather than observable behaviors alone, an interpretive phenomenological approach is methodologically appropriate for examining how educators understand, narrate, and respond to ethical conflict in their professional lives. IPA values idiographic richness over statistical typicality, so prioritizes depth of understanding lived experience over generalisation. As such, teachers’ narratives are foregrounded as testimony to understandings of the impacts of reform, in contestation of officially sanctioned accounts of educational change. IPA was selected specifically because its three epistemological commitments, phenomenology (lived experience as the object of inquiry), hermeneutics (interpretation of participant meaning-making), and idiography (priority to the particular case before cross-case patterning), directly fit the analytic demands of studying moral injury, which is constitutively about how individuals interpret and narrate transgressive ethical events within their particular cultural worlds [43,44]. The final sample of N = 25 sits at the upper end of the range recommended for IPA doctoral and professional studies [44,45], chosen deliberately to permit cross-case thematic patterning across three contrasting regions while preserving idiographic depth within each case.

### Research setting and participants

Data were collected from a national sample of Saudi public-school teachers recruited across multiple regions of the Kingdom. Three primary anchor sites: Riyadh, Jeddah and Abha, provided the initial recruitment access points and represent the three contrasting reform ecologies that frame the analysis. These three anchor sites were purposively selected on theoretical rather than purely geographic grounds, to capture variation in administrative intensity, urban diversity, and community-embedded schooling context. Snowball sampling extended recruitment from each anchor site to surrounding and additional regions, producing a national sample that captures the wider geographic and institutional diversity of Saudi public education under Vision 2030 (see Table 2 for the full geographic distribution; detailed participant characteristics are provided in S1 Table). Participants were Saudi public-school teachers (N = 25) teaching in intermediate and secondary schools, both male and female, across a range of subjects. 32 teachers were approached; seven were eliminated due to incomplete interviews or withdrawal and/or enrolment prior to interview completion, yielding a final analytic sample of 25 participants. Each interview was uniformly transcribed verbatim, and rich idiographic analyses were conducted in parallel across cases as guided by IPA [46]. The sample size was chosen in such a way as to allow for in-depth idiographic ‘diving in’, while still leveraging the potential for richer cross case thematic pattering. Teachers were approached using maximum variation sampling for maximal diversity across gender, career stage (novice, mid-career, veteran), school type (boys’ and girls’ schools; urban and semi-urban) and subject specialization (notably English, science and Islamic studies). Sample recruitment was accomplished by networking with teachers, professional networks, school supervisors, and social networking snowball sampling. Maximum variation sampling was applied deliberately to counteract the clustering that recruitment through professional networks and snowball referral can introduce, recognising that this strategy mitigates rather than eliminates the tendency toward a socially and professionally connected sample. The three regions were selected as anchor sites on theoretical rather than purely geographical grounds, to capture variation in the ecology of reform implementation. Riyadh, as the administrative and political capital, was selected to represent the site of highest-intensity contact with Ministry oversight, inspection regimes, and KPI-driven accountability. Jeddah, as a cosmopolitan coastal metropolis with dense international and private-sector educational influence, was selected to represent urban-diverse reform ecology where global accountability logics meet pluralistic student populations. Abha, as a peripheral region with more strongly community-embedded schooling and stronger continuity with traditional religious-pedagogical practice, was selected to represent reform ecology at greater distance from central administrative intensity. Snowball sampling from each of these anchor sites extended recruitment to additional regions across the Kingdom, allowing the analysis to engage with how a single macrosystem policy is differentially translated and phenomenologically experienced under contrasting institutional-ecological conditions throughout Saudi Arabia.

**Table 2:** Summary of Emergent Themes from Participant Narratives.

Participant (Pseudonyms)	Gender	Location	Years Teaching	Subject	Key Themes
Fatimah	Female	Riyadh	12 years	English	Performance pressure, value conflict, test culture
Ahmed	Male	Jeddah	7 years	Islamic Studies	Ethical conflict, spiritual dissonance, institutional silence
Layan	Female	Abha	15 years	Science	Burnout, identity loss, datafication
Salman	Male	Taif (rural)	20 + years	Arabic Literature	Autonomy loss, systemic betrayal, compartmentalization
Noura	Female	Dammam	2 years	Math	Early career trauma, lack of support, collapse
Rami	Male	Makkah	10 years	Social Studies	Cultural erosion, testing hegemony, less agency
Maha	Female	Tabuk	6 years	Biology	Mentorship loss, rigidity of policy, stagnation
Yousef	Male	Yanbu	11 years	Computer Science	Technostress, depersonalization, role ambiguity
Areej	Female	Al-Ahsa	8 years	History	Curriculum mismatch, heritage loss, emotional fatigue
Khalid	Male	Buraidah	14 years	Chemistry	Top-down constraints, fear of inspection, professional disillusionment
Dina	Female	Hail	9 years	Geography	Civic alienation, routinization, moral dissonance
Tariq	Male	Jizan	5 years	Physics	Pressure between innovation and standardization
Sahar	Female	Najran	13 years	English	Compression of curriculum, rapid provision of instruction, moral tension
Faisal	Male	Qassim	16 years	Islamic Studies	Distrust of religious autonomy, overreach of bureaucracy
Najwa	Female	Hofuf	3 years	Math	Post-tenure insecurity, lack of pedagogical mentorship
Bassam	Male	Al-Kharj	18 years	Arabic	Professional purpose crisis, adaptation fatigue, nostalgic dissonance
Huda	Female	Al-Baha	1 year	Science	Mode of survival, emotional numbing, lack of voice
Osama	Male	Arar	17 years	English	Digital compliance, self-censorship
Reem	Female	Sakaka	4 years	Physics	Fragmented support, isolate emotional, vulnerable
Ziad	Male	Al-Madinah	19 years	Economics	Economic pressure, performance facade, withdrawal
Layla	Female	Khobar	7 years	Art & Design	Creative expression suppression, standardization pressure, cultural identity conflict
Omar	Male	Abha	12 years	Physical Education	Health vs. performance metrics, student wellbeing neglect, time allocation stress
Nora	Female	Riyadh	4 years	Special Education	Inclusion policy gaps, resource constraints, individualized care vs. standardization
Fahad	Male	Al-Jubail	16 years	Business Studies	Market-driven curriculum, ethical commerce teaching, vocational vs. academic tension
Amal	Female	Al-Madinah	9 years	Psychology	Mental health awareness vs. stigma, counseling role expansion, professional boundary confusion

### Data collection

Primary data were obtained through semi-structured in-depth interviews (60–90 min) in-person or via a secure online video tool based on participant location and preference. Interviews were conducted in Arabic or English, depending on participant comfort; transcripts in Arabic were translated and re-checked for conceptual fidelity. Questions explored experiences of ethical conflict, institutions’ pressures, emotional responses to reform, and perceived sources of support. In addition, a small number of participant (n = 10) completed brief reflective journals to capture recurring ethical dilemmas and emotional dilemmas across their daily practice over two weeks; reflections were only used to help situate the interview data, rather than as analytic units in their own right.

### Translation and conceptual equivalence

Arabic-language interviews were transcribed verbatim by a bilingual Saudi research assistant and then independently forward-translated into English by the lead researcher. A second bilingual academic with linguistic and educational research expertise back-translated a 20% sample of the translated transcripts into Arabic; discrepancies were resolved through iterative discussion with attention to connotative and culturally specific terminology (for example, the religious semantic fields of ‘*Muraqib*’, ‘*Amanah*’, and ‘*Rahmah*’, which do not map cleanly to single English terms). Culturally embedded concepts were retained in transliterated Arabic in the final analytic transcripts with interpretive glosses, to preserve phenomenological specificity and reduce the risk of meaning loss in translation.

### Data analysis

Data were analysed according to IPA approaches [42]. Transcripts were read multiple times to get a sense of emotional tenor and narrative arc. Exploratory notes were generated. Themes were then generated both within case, and across all cases, to attend to shared felt-meanings while retaining idiographic specificity. Inductive coding addressed emergent concepts present in participants’ narratives. Sensitizing concepts from the interview literature on moral injury, including betrayal, moral dissonance, and systemic coercion, informed the analyses but did not pre-specify coding categories. These sensitizing frameworks served as a tool for interpretation, but no categories were defined in advance, with themes being developed from the participants themselves. Serial re-readings of transcripts were done to attend to tone, narrative arc, and culturally patterned language. Exploratory notes were created at three “levels”: descriptive (what is actually said), linguistic (how it is said), and conceptual (what it might indicate); with particular attention to instances where institutional expectations potentially transgressed teachers’ professional and moral commitments. Emergent themes were developed within-case before patterning across cases. Coding was done in such a way as to marry inductive emergence into themes from participants’ accounts with theoretically sensitized attention to concepts from the literature on moral injury such as ideas of betrayal, moral dissonance, constrained agency. Analysis proceeded through the six-stage protocol articulated by Smith, Flowers and Larkin [43] and subsequently elaborated by Pietkiewicz and Smith [44] and Larkin et al. [42]: (1) multiple immersive readings of each transcript to grasp narrative arc and emotional tenor; (2) three-level exploratory noting (descriptive, linguistic, conceptual) in the transcript margins, with particular attention to moments where institutional demand appeared to transgress professional or moral commitment; (3) development of emergent themes from these notes, staying close to participant language; (4) searching for connections among emergent themes within each case to generate a structured set of super-ordinate themes; (5) repetition of stages 1–4 for each remaining case, preserving idiographic priority and guarding against premature cross-case imposition; and (6) cross-case patterning to identify shared super-ordinate themes while retaining individual variation. Sensitizing concepts drawn from the moral injury literature, betrayal, moral dissonance, constrained agency informed interpretation at stages 2 and 3 without pre-specifying coding categories.

### Trustworthiness and rigor

To enhance reliability and trustworthiness, multiple strategies were utilized. Participant feedback was obtained through member checking when the participants were given opportunities to read excerpts from selected transcripts and provide initial comments regarding the interpretation of those excerpts. The use of a bilingual academic peer for debriefing sessions facilitated an increase in reflexivity and analytical credibility with regards to the Saudi Arabian context. The researcher established an audit trail for tracking analytical decisions and developing themes; additionally, the researcher maintained a reflexive journal throughout the analysis to explore their positionality and emotional reaction at different points throughout the study. Rigor was operationalized explicitly against Lincoln and Guba’s [47] four criteria. Credibility was supported through prolonged engagement with participants (60–90 minute interviews with follow-up clarification where needed), member-checking of selected interpretive excerpts by ten participants, and peer debriefing with a bilingual academic familiar with the Saudi educational context. Dependability was supported through an analytic audit trail documenting each decision from raw transcript to final super-ordinate theme, retained under the institutional data-governance arrangements described in the Data Availability Statement. Confirmability was supported through the researcher’s reflexive journal, in which positionality, emotional reactions during analysis, and potentially biasing commitments were recorded and revisited at the theme-consolidation stage. Transferability is addressed through thick contextual description of the three anchor research settings, of the wider regional spread of the sample, and of each participant, inviting readers to assess analytic fit to their own contexts rather than relying on statistical generalization.

### Ethical considerations

Ethics approval was granted by the Research Unit at the English Language Institute, King Abdulaziz University (IRB #2025-EDU-201, approved 25 April 2025), before data collection commenced. All participants consented to having been provided with a description of the purpose of the study, how confidentiality would be maintained, and their ability to withdraw from the study without penalty, both orally and in writing prior to commencing their participation. De-identified data are stored securely on encrypted, password-protected institutional systems and may be accessed only through the controlled institutional process described in the Data Availability Statement. Given the sensitive nature of ethical reflection, interviews were conducted using trauma-informed practices, and participants were free to pause or terminate interviews at any time.

### Limitations

As an idiographic qualitative study, findings are not intended to be statistically generalizable but are offered for analytical transferability through contextualized description. Cultural norms surrounding emotional expression may have influenced the degree of disclosure among some participants. In addition, the study reflects educators’ perspectives without parallel institutional viewpoints and therefore captures only part of the broader reform ecology influencing teacher wellbeing. There are five key limitations of the study that should be defined as such. The first limitation is that the findings presented are based only on teacher’s perspectives. Therefore, access was not available to the perspectives of Ministry officials, school administrators and reform architects who might have provided added context to the moral injury framing in some circumstances. The second is the fact that the sample, although drawn from multiple regions across Saudi Arabia and anchored in three primary sites (Riyadh, Jeddah, and Abha) selected on theoretical grounds, does not claim full representational coverage of the Kingdom; the findings may not apply uniformly to all regions or to private-sector schooling. Third, the possibility that the ethical nature of the interviews selected for participants who were already experiencing some level of distress, led to salience bias and increased the chance of participants presenting negative frames, should be highlighted, as this study does not claim to be representative of the entire population as to how the reforms were experienced. Fourth, while it is not disputed that these reforms may provide wider access to higher levels of technology, improved employability for graduates and an increase in the level of international comparison between the Saudi systems, the findings of the moral injury should not be used to form an overall judgement on the success of the reforms and to represent a specific, mitigable cost associated with the implementation of these reforms. Fifth, because recruitment proceeded through professional networks and snowball referral from the three anchor sites, the sample is likely to over-represent teachers who are socially and professionally connected and who may share broadly similar dispositions toward the reforms, which introduces a risk of homogeneity and selection bias; this recruitment route may have shaped the range of experiences captured, for example by drawing in educators already disposed to articulate ethical distress, and the maximum variation strategy described in the Methods can only partially offset this tendency.

### Data analyses and results

#### Analytic orientation.

This section presents findings derived from interpretative phenomenological analysis (IPA), focusing on how educators make sense of ethical conflict and professional identity under reform conditions. While qualitative interpretation remains central, descriptive summaries are used to illustrate patterns of thematic prominence across cases and to support transparency in theme development. These descriptive indicators do not replace idiographic analysis or imply statistical generalization.

#### Overview of emergent themes.

Analysis identified six interrelated themes reflecting participants’ experiences of reform-related ethical strain: systemic pressures, curriculum rigidity, reduced relational care, value dissonance, burnout, and lack of institutional empathy. Table 2 summarizes participant characteristics and theme occurrence. These summaries provide a structural overview of experiential patterns prior to in-depth thematic interpretation.

### Exosystem pressures: institutional translation of reform

Informants routinely described these reform pressures emerging not so much through direct contact with national policy texts but rather through institutional experience of ministry missives, the inspection cycle, and performance management: “*It’s more through the experienced pressures and routines we go through rather than the policy documents we actually read sometimes*” (Maha). As these exosystemic structures translate macrolevel reform agendas into daily expectations of compliance, they produce intensifications of surveillance, documentation burden, and constraints on authentic instructional practice. Ahmed describes how conversion of time assigned to moral instruction to assessment preparation is “*like sacrificing the soul of education*”. Read closely at the level of language, these two accounts disclose how the exosystem is encountered phenomenologically rather than textually. Maha locates reform not in the documents she reads but in the routines she lives, so that policy is felt as atmosphere and habituated obligation before it is ever cognitively engaged. Ahmed’s choice of the verb sacrificing, drawn from a religious register, reframes what the institution presents as a neutral reallocation of instructional time as the loss of something he regards as central to teaching. At the conceptual level, this is the point at which moral injury originates for these participants: an institutional efficiency is experienced as acting against a deeply held value, with the teacher positioned as the one required to enact it.

### Microsystem conflict: classroom-level ethical dissonance

The demands of institutions were most directly felt through classroom encounters where teachers contended with time constraints, curriculum pacing mandates, and challenges to their relational capacity. Teachers described feeling forced to prioritize test preparation and compliance above individual student support and how these practices produced ethical discomfort and personal betrayal of the teacher identity they wished to enact. These microsystem-level “hot spots” of moral tension surfaced as the central sites of moral dilemmas. A handful of teachers talked about ‘practicing’ the things they found themselves doing in classrooms that provoked a sense of moral conflict in their disposition toward students. “*I’m betraying them.... Even maintaining an 85% performance, regardless of where they are, that’s betraying them*” (Fatimah). Fatimah’s words repay close interpretive attention. Her repeated use of the word betraying, and her placement of herself in the first person as the agent of that betrayal rather than its object, indicate that the moral wound here is one of perceived perpetration rather than of harm suffered at the hands of others. The precise figure of an eighty-five per cent performance target is itself telling, since the metric becomes the very instrument through which she is made to betray students whose needs she continues to perceive clearly. At the conceptual level, the classroom emerges not merely as a site of strain but as the place where a teacher is conscripted into acting against her own ethical commitments, which is the distinctive structure of moral injury as distinct from ordinary occupational stress.

### Individual internalization: moral injury and burnout

Repeated exposure to unresolved ethical conflict was embodied as emotional and identity-related distress. Participants described experiences of feeling betrayed morally, harming an experience of professional day’s work, and suffering a sense of diminished worth. For these participants, burnout was bound up with unresolved moral conflict rather than arising simply from depleted resources, so that moral injury is best understood as an injury to the ethical self. Layan, a science teacher, captured this erosion when she described moving from “*sparking curiosity*”, to a “*data technician*,” and then on medical leave every year due to anxiety. Layan’s two images map a single trajectory of identity loss. The first, sparking curiosity, names a generative and relational vocation in which teaching ignites something in another person; the second, data technician, names a mechanical and depersonalised function in which the teacher merely processes outputs. That she narrates the latter as what she has become, and that the cost recurs in her body as annual medical leave, shows how the ethical conflict described above does not remain situational but settles into a revised understanding of who she is. This is the point at which systemic pressure is fully internalised as moral injury, no longer an external demand but a change in the self.

### Chronosystem differences: career stage and coping patterns

Experiences of moral injury differed at each stage of participants’ careers. Early career teachers were more likely to describe anxiety, fear of non-compliance, or confusion about their professional identity. Veteran teachers were more likely to find ways to accommodate their work, express detachment or metaphorical compartmentalization as a way to remain in their jobs while minimizing the ethical distress. These two chronosystem strands suggest that a teacher’s career stage influences his/her susceptibility to moral injury and also suggests that veteran teachers speak more of adaptive but ethically ambiguous strategies to cope. One senior Arabic teacher described “*performing compliance*” for inspectors and trying to continue to play the authentic teaching role on his own time, a necessary strategy that he also found shameful (Salman). In contrast, early-career teachers described immediate psychological vulnerability. One novice teacher entering the profession during reform implementation reported panic attacks related to KPI expectations and feelings of institutional disposability (Noura). The contrast between these two cases is itself analytically revealing. Salman’s phrase performing compliance casts his working life in dramaturgical terms, splitting the self into a compliant public performance staged for inspectors and an authentic practice reserved for his own time. That he describes this division as shameful indicates that the strategy does not resolve the moral conflict so much as relocate it inward, so that veteran adaptation is better read as a managed and ongoing wound than as recovery. Noura, by contrast, has no comparable private space into which to retreat; her panic attacks and her sense of institutional disposability show the injury in its acute and bodily form, before the career-long strategies of compartmentalisation that Salman has built up are available to her. Read together, the two accounts suggest that career stage shapes not whether moral injury is experienced but the form its concealment or expression takes.

### Descriptive patterns of thematic salience

Across the 25 cases, the super-ordinate themes differed in salience, understood here as the combination of how widely a theme recurred across participants and how centrally it featured within individual accounts. Some themes were pervasive, surfacing in most narratives and often articulated with considerable depth, while others, though clearly present, appeared in fewer accounts or with less emphasis. This patterning was established interpretively through the idiographic analysis rather than through any numerical index, and the qualitative descriptions that follow convey the relative prominence of each theme without reducing it to a score.

### Summary of results in relation to the ecological model

Collectively, findings are broadly consistent with the proposed ecological moral injury framework, which is advanced here as an interpretive and heuristic account derived abductively from a small purposive sample rather than as an empirically validated or generalizable model, in which macro-level reform agendas are translated through institutional mechanisms, enacted through classroom constraints, and internalized as ethical and identity-related harm. Participants’ narratives illustrate how systemic governance structures shape moral experience, supporting the interpretation that moral injury among educators is structurally produced rather than individually generated.

### Interpretive Insight

From the data emerged several themes across cases: (1) moral dissonance from policies; (2) spiritual dilemmas and loss of integrity as a teacher; (3) emotional exhaustion and burnout; (4) decreased autonomy; and (5) disconnection with the leaders of the organization. These themes bring to light systemic ethical tensions related to the implementation of Vision 2030.

## Discussion

The results of the study suggest that Saudi educators may be suffering moral injury in the form of ethical dissonance, emotional distress and discontentment with one’s professional identity as a consequence of being expected to implement Vision 2030 reforms. Creating an accountability regime supported by punishment and rewards is a governmental policy concern, but one must consider whether the resulting “blame and guilt game” led to morally injurious psychological outcomes for teachers whose classrooms were core sites of this reform [48]. Litz et al. [10] emphasized that morally injurious harm is not necessarily in the form of individual vulnerability but rather, is the internalization of a system’s failure to prevent an injustice”.

Findings must be positioned in the context of an evaluation which evaluates Vision 2030 objectively. The reform programme provided a documented increase, improvement in curriculum modernisation, enabling more women to participate in STEM, greater international comparability of educational outcomes, and a major financial commitment to educational technology; and that participants in this study acknowledged the importance of those benefits along with their experiences of distress. The central proposition of this paper is that Vision 2030 does not have a negative impact on aggregate perspectives, but the mechanism for implementing Vision 2030, including KPI (Key Performance Indicator) driven pacing, the introduction of inspection regimes and stylised systems for the measurement of compliance with common standards, creates specific ethical costs to teachers and this has not been sufficiently acknowledged in the current discourse about reform and there is scope for addressing the problem of the experiences of moral injury while at the same time maintaining the developmental aspirations of the reform programme. In this sense, the recognition of the issue of moral injury adds to, rather than detracts from the developmental aspirations of the reform programme.

### From reform agents to moral subjects

Participants’ accounts reflect a shift from being positioned as professional reform agents to becoming moral subjects navigating ethical compromise. Educators described feeling betrayed not by individuals but by institutional structures that prioritize compliance over care, consistent with international findings that link accountability regimes to teacher demoralisation rather than burnout alone [7,13]. This experience was further heightened through cultural and religious context in Saudi Arabia. Teachers experienced professional frustrations and spiritual and ethical ruptures when institutional mandates conflicted with the Islamic principles of justice, equity, and mercy, which are utilized in teaching as a form of moral stewardship.

In addition, Islamic Studies and Arabic language teachers expressed being forced to put more emphasis on assessment preparation than on the development of reflective and values-based learning, which detracted from the ethical intent of their teaching. In kind, teachers of English and science in under-resourced classroom settings reported experiencing moral conflict wherein the use of standardized curricula did not match their students’ academic or linguistic readiness. Across all cases, educators expressed feeling complicit in the educational harm, experiences which parallel key characteristics of moral injury.

### Curriculum rigidity and datafication

Participants frequently described experiencing data-driven accountability as transforming teaching into a form of labour increasingly organised around metrics and compliance, which they articulated in terms that resonate with accounts of neoliberal governance’s appropriation of professional judgement into a technical compliance standard [49]. Teachers’ description of themselves as “data technicians” instead of educators conveyed a sense that their professional agency and the ethical relationships they held with their students had eroded. While some teachers have adapted their teaching in covert ways in order to superficially satisfy the requirements for compliance, they continue to actually teach in a way that is authentic within the context of their practice. These covert means of adaptation and compliance have an ethical cost that perpetuates distrust of institutions and moral compromises, rather than addressing the systemic tensions arising from this relationship between teaching and data-driven accountability.

### Chronosystem effects and differential vulnerability

Career-stage differences observed in this study are consistent with extending the interpretive framework to include the chronosystem. Early career teachers reported acute anxiety, identity instability, and doubts about professional longevity, compounded by limited mentorship and institutional support. Veteran teachers more often employed coping strategies such as compartmentalization and selective compliance, which reduced immediate psychological distress but did not eliminate ethical conflict. These findings suggest that professional experience moderates exposure and response to moral injury but does not shield educators from its structural origins. Differentiated support mechanisms may therefore be valuable, particularly for novice teachers lacking institutional capital and reflective professional networks.

### Institutional empathy and moral voice

A pervasive absence of institutional empathy emerged across participant accounts. Teachers described limited opportunities to express ethical concerns or participate meaningfully in reform discourse, reinforcing perceptions of disposability and institutional betrayal. International research highlights that teacher wellbeing is closely tied to perceived fairness, participatory governance, and alignment between institutional and personal values [50]. In the Saudi reform context, opaque decision-making processes and rigid implementation pathways appear to have generated a trust deficit, intensifying moral disengagement and attrition risk.

### Implications for policy and professional practice

The findings suggest that sustainable reform requires attention not only to instructional outcomes but also to educators’ moral and relational realities. The recommendations set out in this section are interpretive proposals that extend beyond the study’s empirical findings; they are offered provisionally, in light of the limitations noted above, rather than as conclusions established by the data. If Vision 2030 seeks to balance global competitiveness with cultural integrity, reform design must incorporate ethical responsiveness alongside performance accountability. The primary practical implications of the study can be addressed through five specific, implementable actions: (1) ethical-impact assessment of new KPI and pacing policies prior to their implementation, analogous to health-impact assessment in public policy; (2) formalised protected spaces for the expression of teacher ethical voice as part of school governance, for example through the establishment of a standing pedagogical ethics committee with substantive advisory authority; (3) moral-injury awareness training for school leaders and inspection personnel to help them recognise and avoid inflicting ethical harm on teachers; (4) differentiated mentorship structures designed to address the unique needs of early-career teachers identified in this study; and (5) redesigned accountability indicators that incorporate the ethical and relational quality of the institution, for example, teacher perceptions of alignment between institutional practice and religious/pedagogical values, alongside test-performance metrics.

Recognizing moral injury as a systemic rather than individual phenomenon shifts reform responsibility from teacher resilience toward institutional design. For many participants in this study, educational transformation was experienced not solely as infrastructural or curricular change but as a morally consequential process. Without ethical alignment between policy and practice, reform risks undermining the very professional commitment it seeks to mobilise.

## Conclusion

This study complements on emerging work on teacher moral injury by conceptualising ethical distress in relation to its cultural and policy context, here the ongoing Vision 2030 reforms in Saudi Arabia. As in much comparative work, teachers’ suffering in this study was not so much related to the burden of workload and emotional exhaustion, but to experiences of betrayal, curtailed moral agency and compulsion to values in conflict/juxtaposition resulting from accountability-oriented school reform. In the Saudi context, however, teachers are themselves required to present their work as moral stewardship. Where institutional requirements conflict with Quranic exhortations of pedagogic justice and care, this ethical dissonance does not simply manifest as frustration in the workplace but destabilizes the pedagogue’s spiritual and social identity. By linking moral injury theory with Bronfenbrenner’s ecological systems framework, the findings suggest that moral injury may manifest through interactions among systemic layers rather than through single classroom events. In these participants’ accounts, policy priorities were translated through institutional and constraining mechanisms, voiced in terms of constraint in the classroom, and reduced the individual teacher to ethical and psychological harm, and career stage moderated both risk and sensemaking. That moral injury constitutes a socially and structurally ‘produced’ condition rather than ‘failure’ of the individual suggests, for the educators in this study, that responses to teacher wellbeing may need to be institutional rather than solely individual. These assertions are made with due epistemological humility. In the present study, the analytic rather than the statistically generalisable contribution is made, and the ecological moral injury framework is offered as a culturally specific and interpretive account open for further testing and refinement (and, it is hoped, improvement further) in other Islamic and non-Western educational settings rather than as a coherent universal account.

This work contributes to international debates by interrogating empirical material from a non-Western, religiously situated education system, a perspective largely absent from the moral injury literature. As education reforms accelerate across the Gulf region and the broader Middle East and Asia, understanding the entanglement of models of governance circulating internationally with local moral traditions grows increasingly pertinent. The accounts in this study suggest that, where policy orientation and practice lack ethical congruence, reform may risk weakening the professional commitment and relational trust that these participants described as central to their work. While this work sheds light on teachers’ lived experience, it does not capture the experiences of school leaders or institutional decision-makers, nor does it speak directly to ethical questions that teachers occupy. Future work that centres on leadership and policy-level ‘readings’ of reform ethics norms, that produces instruments for the (careful and contextualised) measurement of moral injury in line with local cultural interests, and that strives for longitudinal designs are of particular importance in relation to understanding how moral injury is (re)negotiated across professional trajectories, and how moral injury informs teacher retention.

## Supporting information

S1 Table Detailed participant characteristics including career stage, subject, school type, and regional setting. (DOCX)
