# Supplementary material for: Moral injury and burnout among saudi educators under vision 2030: An interpretative phenomenological study of systemic ethical tensions
Source: PLoS One. 2026 Aug 14;21(8):e0356254. doi: 10.1371/journal.pone.0356254 (PMC13475912; doi:10.1371/journal.pone.0356254)
Supplement: Supplementary file 1 [file pone.0356254.s001.docx]

**S1 Table.** Detailed characteristics of all participants (N = 25).

*Note. All participant names are pseudonyms. Maximum-variation sampling was used to ensure diversity across gender, career stage, school type, subject specialization, and region, with three primary anchor sites in Riyadh, Jeddah, and Abha and snowball recruitment extending across multiple Saudi regions.*

| **Pseudonym** | **Gender** | **Age Range** | **Region** | **School Type** | **Subject Specialization** | **Career Stage** | **Years of Experience** | **Highest Qualification** |
| --- | --- | --- | --- | --- | --- | --- | --- | --- |
| Huda | F | 20–30 | Al-Baha | Public | Science | Early | 1 | Bachelor’s |
| Fatimah | F | 30–40 | Riyadh | Public | English | Mid | 12 | Master’s |
| Ahmed | M | 30–40 | Jeddah | Public | Islamic Studies | Mid | 7 | Master’s |
| Salman | M | 45–55 | Taif (rural) | Public | Arabic Literature | Late | 20+ | Master’s |
| Layan | F | 40–50 | Abha | Public | Science | Mid | 15 | Master’s |
| Noura | F | 20–30 | Dammam | Public | Mathematics | Early | 2 | Bachelor’s |
| Rami | M | 30–40 | Makkah | Public | Social Studies | Mid | 10 | Bachelor’s |
| Maha | F | 30–40 | Tabuk | Public | Biology | Mid | 6 | Master’s |
| Yousef | M | 30–40 | Yanbu | Public | Computer Science | Mid | 11 | Bachelor’s |
| Areej | F | 30–40 | Al-Ahsa | Public | History | Mid | 8 | Master’s |
| Khalid | M | 35–45 | Buraidah | Public | Chemistry | Mid | 14 | Master’s |
| Dina | F | 30–40 | Hail | Public | Geography | Mid | 9 | Bachelor’s |
| Tariq | M | 25–35 | Jizan | Public | Physics | Mid | 5 | Bachelor’s |
| Sahar | F | 35–45 | Najran | Public | English | Mid | 13 | Master’s |
| Faisal | M | 40–50 | Qassim | Public | Islamic Studies | Late | 16 | Master’s |
| Najwa | F | 20–30 | Hofuf | Public | Mathematics | Early | 3 | Bachelor’s |
| Bassam | M | 45–55 | Al-Kharj | Public | Arabic | Late | 18 | Bachelor’s |
| Osama | M | 40–50 | Arar | Public | English | Late | 17 | Bachelor’s |
| Reem | F | 25–35 | Sakaka | Public | Physics | Early | 4 | Bachelor’s |
| Ziad | M | 45–55 | Al-Madinah | Public | Economics | Late | 19 | Master’s |
| Layla | F | 30–40 | Khobar | Public | Art & Design | Mid | 7 | Bachelor’s |
| Omar | M | 30–40 | Abha | Public | Physical Education | Mid | 12 | Bachelor’s |
| Nora | F | 20–30 | Riyadh | Public | Special Education | Early | 4 | Bachelor’s |
| Fahad | M | 40–50 | Al-Jubail | Public | Business Studies | Late | 16 | Master’s |
| Amal | F | 30–40 | Al-Madinah | Public | Psychology | Mid | 9 | Master’s |

*Career Stage bands: Early-career = fewer than 5 years of teaching; Mid-career = 5–15 years; Late-career = more than 15 years. Highest Qualification: B.Ed./BA = Bachelor's; M.Ed./MA = Master's; Ph.D. = Doctorate.*
